# Preoperative FAN score predicts survival outcomes after radical cystectomy for bladder cancer

**DOI:** 10.1002/bco2.70221

**Published:** 2026-04-30

**Authors:** Yu Ishizuya, Atsunari Kawashima, Shimpei Yamashita, Takeshi Ujike, Tetsuya Takao, Osamu Miyake, Hiroki Osaki, Hiromu Horitani, Toshichika Iwanishi, Tsuyoshi Takada, Yuta Oki, Takahiro Yoshida, Makoto Matsushita, Ken‐Ichi Kakimoto, Akira Nagahara, Takuji Hayashi, Yoshiyuki Yamamoto, Taigo Kato, Koji Hatano, Yasuo Kohjimoto, Isao Hara, Norio Nonomura

**Affiliations:** ^1^ Department of Urology The University of Osaka Graduate School of Medicine Suita Japan; ^2^ Department of Urology Wakayama Medical University Wakayama Japan; ^3^ Department of Urology Osaka Rosai Hospital Sakai Japan; ^4^ Department of Urology Osaka General Medical Center Osaka Japan; ^5^ Department of Urology Toyonaka Municipal Hospital Toyonaka Japan; ^6^ Department of Urology Osaka Keisatsu Hospital Osaka Japan; ^7^ Department of Urology Higashiosaka City Medical Center Higashiosaka Japan; ^8^ Department of Urology Sakai City Medical Center Sakai Japan; ^9^ Department of Urology Minoh City Hospital Minoh Japan; ^10^ Department of Urology National Hospital Organization Osaka National Hospital Osaka Japan; ^11^ Department of Urology Hyogo Prefectural Nishinomiya Hospital Nishinomiya Japan; ^12^ Department of Urology Ikeda Municipal Hospital Ikeda Japan; ^13^ Department of Urology Nippon Life Hospital Osaka Japan; ^14^ Department of Urology Osaka International Cancer Institute Osaka Japan

**Keywords:** ALBI score, bladder cancer, fib‐4 index, NLR, prognostic factor, radical cystectomy

## Abstract

**Objective:**

This study aimed to evaluate whether the preoperative FAN score—composed of the fibrosis‐4 (Fib‐4) index, albumin–bilirubin (ALBI) score and neutrophil–lymphocyte ratio (NLR)—predicts recurrence‐free, cancer‐specific and overall survival after radical cystectomy for bladder cancer.

**Patients and Methods:**

We retrospectively analysed 1121 patients who underwent radical cystectomy at 13 institutions between April 2010 and March 2024. Associations between the FAN score and recurrence‐free survival (RFS), cancer‐specific survival (CSS) and overall survival (OS) were evaluated. Prognostic performance was assessed in an independent cohort of 296 patients from three institutions.

**Results:**

FAN score distribution was 0 (*n* = 600, 53.5%), 1 (*n* = 409, 36.5%) and ≥2 (*n* = 112, 10.0%). Patients with a FAN score ≥2 had significantly worse RFS (median: not reached vs 12.3 months; *p* < 0.0001), CSS (not reached vs 22.8 months; *p* < 0.0001) and OS (112.5 vs 16.1 months; *p* < 0.0001) than those with a FAN score ≤1. On multivariable analysis, a FAN score ≥2 was an independent predictor of poorer RFS (HR 2.12, 95% CI 1.55–2.91; *p* < 0.0001), CSS (HR 2.80, 95% CI 2.00–3.92; *p* < 0.0001) and OS (HR 2.70, 95% CI 2.03–3.59; *p* < 0.0001). These associations were consistently observed in an independent cohort of 296 patients.

**Conclusions:**

The FAN score is an independent prognostic marker of adverse outcomes after radical cystectomy for bladder cancer and may help stratify patients for perioperative management and follow‐up.

## 1 INTRODUCTION

Radical cystectomy (RC) is the standard treatment for muscle‐invasive bladder cancer.^1^, ^2^ However, RC is highly invasive and often reduces patients' quality of life because of urinary diversion. Some patients experience early recurrence after RC, leading to poor long‐term survival and outcomes that may not justify the procedure's invasiveness. Therefore, identifying patients at high risk of poor prognosis before RC is essential. Reported preoperative prognostic factors include clinical stage,^1^ hydronephrosis,^3^ vascular invasion,^4^ histological variants in TURBT specimens,^5^ serum albumin levels,^6^ anaemia,^7^ age^8^ and performance status (PS).^9^ We previously showed that the FAN score—composed of the Fib‐4 index, ALBI score and neutrophil–lymphocyte ratio (NLR)—predicts outcomes in metastatic urothelial carcinoma treated with first‐line platinum‐based chemotherapy^10^ and with second‐line pembrolizumab.^11^ The FAN score can be calculated easily from routine blood tests and does not require special or invasive procedures. The aim of this study was to determine whether the FAN score predicts prognosis in patients with muscle‐invasive bladder cancer undergoing RC.

## 2 PATIENTS AND METHODS

### 2.1 Patient cohort and treatment

We retrospectively enrolled 1121 patients who underwent RC for bladder cancer between April 2010 and March 2024 at 13 institutions (Osaka University Hospital and affiliated hospitals; listed by number of registered patients: Osaka Rosai Hospital; Osaka General Medical Center; Toyonaka Municipal Hospital; Osaka Police Hospital; Higashiosaka City Medical Center; Sakai City Medical Center; Minoh City Hospital; National Hospital Organization Osaka National Hospital; Hyogo Prefectural Nishinomiya Hospital; Ikeda Municipal Hospital; Nippon Life Hospital; Osaka International Cancer Institute). Patients with clinical distant metastasis (cM+) at the time of RC were excluded from the analysis. An independent cohort comprised 296 patients who underwent RC at Wakayama Medical University and its affiliated hospitals.

### 2.2 Data collection

Patient information prior to RC was used for analysis. Information on age, gender, body mass index (BMI), Eastern Cooperative Oncology Group performance status (ECOG PS), clinical TNM stage, neoadjuvant chemotherapy, prior surgery for upper tract urothelial carcinoma, serum albumin, serum bilirubin, serum aspartate aminotransferase (AST), serum alanine aminotransferase (ALT), estimated glomerular filtration rate (eGFR),^12^ neutrophil count and lymphocyte count were collected from medical records. The fibrosis‐4 (Fib‐4) index has been reported as an indicator of liver fibrosis and is calculated by the following formula: age (years) × AST (U/L) / (platelet count [10^3^/μL] × (ALT [U/L]^0.5^).^13^ The albumin–bilirubin (ALBI) score is a measure of liver function that can be calculated using the following formula: log_10_ (T‐bil [mg/dL] × 17.1) × 0.66 + albumin [mg/dL] × 10 (− 0.085).^14^ The neutrophil–lymphocyte ratio (NLR) was calculated using the following formula: neutrophil count/lymphocyte count. Cutoff values for the following parameters were determined based on previous reports: Fib‐4 index > 3.5,^15^ ALBI score > − 2.6^14^ and NLR > 5.0.^16^ The FAN score was defined as the number of the three factors (Fib‐4 index > 3.5, ALBI score > − 2.6 and NLR > 5.0) that were met (ranging from 0 to 3 points), with a cutoff of ≥2 points, as in our previous report.^10^, ^11^

Recurrence‐free survival (RFS) was defined as the interval from the date of RC to radiological or clinical recurrence or last follow‐up. Cancer‐specific survival (CSS) and overall survival (OS) were defined as the intervals from the date of RC to death from bladder cancer and death from any cause, respectively, or last follow‐up.

### 2.3 Statistical analysis

Survival curves for RFS, CSS and OS were estimated using the Kaplan–Meier method and compared with the log‐rank test. Associations between clinical variables (including FAN score) and survival outcomes were assessed using Cox proportional hazards models. Age was included as a continuous variable in all multivariable Cox models, and clinical T stage was categorised as ≤cT1, cT2 and ≥cT3. All statistical analyses were performed with JMP Pro version 17.0 (SAS Institute Inc., Cary, NC). Two‐tailed *p*‐values < 0.05 were considered statistically significant.

## 3 RESULTS

### 3.1 Patient characteristics

The cohort included 1121 patients who underwent RC. Median age was 73 years (range 29–90). Eight hundred forty‐seven patients (75.6%) were male, and 274 (24.4%) were female. Ninety‐two patients (8.2%) had a history of surgery for upper‐tract urothelial carcinoma (UTUC), and 531 patients (47.4%) received neoadjuvant chemotherapy. Three hundred fifty‐seven patients (31.9%) had clinical T stage ≥3, and 90 patients (8.0%) had clinical N stage ≥1. Surgical approach was open in 417 patients (37.2%), laparoscopic in 195 (17.2%) and robot‐assisted in 509 (45.4%). Patients with Fib‐4 > 3.5, ALBI > − 2.6 and NLR > 5.0 numbered 58 (5.2%), 446 (39.8%) and 138 (12.3%), respectively. FAN score distribution was 0 in 600 cases (53.5%), 1 in 409 (36.5%), 2 in 103 (9.2%) and 3 in 9 (0.8%). Median follow‐up was 32.3 months (range 0.5–174.9) (Table 1).

**TABLE 1:** Patient characteristics.

Characteristic	*n* = 1121	
Age, years, range, (median)	29–90	73.2
Gender, *N* (%)		
Male	847	75.6
Female	274	24.4
BMI, kg/m^2^, range, (median)	13.4–38.5	22.0
ECOG PS, *N* (%)		
0	534	47.6
1	471	42.0
≥2	116	10.3
Prior surgery for UTUC, *N* (%)		
No	1029	91.8
Yes	92	8.2
Neoadjuvant chemotherapy, *N* (%)		
No	590	52.6
Yes	531	47.4
eGFR (ml/min/1.73 m^2^), range, (median)	3–177	57
eGFR (ml/min/1.73 m^2^), *N* (%)		
>60	497	44.3
30–60	529	47.2
<30	95	8.5
Fib‐4 index, *N* (%)		
<3.5	1063	94.8
≥3.5	58	5.2
ALBI score, *N* (%)		
≤−2.6	675	60.2
>−2.6	446	39.8
NLR, *N* (%)		
<5.0	983	87.7
≥5.0	138	12.3
FAN score, *N* (%)		
0	600	53.5
1	409	36.5
2	103	9.2
3	9	0.8
Histopathology of TURBT specimens, *N* (%)		
Urothelial carcinoma	931	83.1
Urothelial carcinoma with variant	147	13.1
Other histology	43	3.8
Clinical T stage, *N* (%)		
0	4	0.4
is	126	11.2
a	20	1.8
1	110	9.8
2	504	45.0
3	260	23.2
4	97	8.7
Clinical N stage, *N* (%)		
0	1031	92.0
1	54	4.8
2	32	2.9
3	4	0.4
Radical cystectomy procedure, *N* (%)		
Open	417	37.2
Laparoscopic	195	17.4
Robot‐assisted	509	45.4
Lymph node dissection		
No	115	10.3
Yes	1006	89.7
Observation period, month, range (median)	0.5–174.9	32.3

Abbreviations: ALBI, albumin–bilirubin; ECOG PS, Eastern Cooperative Oncology Group performance status; eGFR, estimated glomerular filtration rate; Fib‐4, fibrosis‐4; NLR, neutrophil‐lymphocyte ratio; TURBT, transurethral resection of bladder tumour; UTUC, upper tract urothelial carcinoma.

### 3.2 Comparison of clinicopathological factors between the FAN score groups

Table S1 compares 1009 patients with FAN score ≤1 and 112 patients with FAN score ≥2. The FAN ≥2 group was older (*p* < 0.001), had worse PS (ECOG PS; *p* < 0.001), a lower proportion receiving neoadjuvant chemotherapy (*p* = 0.001), lower eGFR (*p* = 0.001) and higher clinical T stage (*p* < 0.001). There were no significant differences between groups for sex, BMI, TURBT histopathology or clinical N stage.

### 3.3 Survival outcomes

Kaplan–Meier survival curves for the full cohort are shown in Figure S1. Median OS was 101.9 months (95% CI 81.5–122.4). Median RFS and CSS were not reached.

### 3.4 Multivariable analysis of factors associated with survival

In multivariable Cox models, FAN score ≥2 was an independent predictor of worse RFS (HR 2.12, 95% CI 1.55–2.91; *p* < 0.0001) along with ECOG PS ≥ 2 (HR 1.70, 95% CI 1.22–2.39; *p* = 0.002), prior UTUC surgery (HR 1.47, 95% CI 1.02–2.13; *p* = 0.041), higher clinical T stage (cT2: HR 1.92, 95% CI 1.39–2.66; ≥cT3: HR 2.82, 95% CI 2.00–3.96; both *p* < 0.0001) and higher clinical N stage (HR 1.67, 95% CI 1.21–2.30; *p* = 0.002) (Table 2).

**TABLE 2:** Univariable and multivariable Cox regression analysis of RFS.

	Univariable				Multivariable			
	HR	95% CI		*P*‐value	HR	95% CI		*P*‐value
		Lower	Higher			Lower	Higher	
Age								
Continuous (/1 year)	1.01	0.99	1.02	0.360	0.99	0.98	1.01	0.542
Gender								
Male	Reference			—	Reference			—
Female	0.94	0.75	1.19	0.624	0.83	0.65	1.04	0.108
BMI								
Continuous (/1 kg/m^2^)	0.97	0.94	0.99	0.033	0.97	0.94	1.00	0.053
ECOG PS								
0	Reference			—	Reference			—
1	1.06	0.86	1.31	0.590	1.01	0.81	1.26	0.924
>=2	1.87	1.36	2.58	<0.001	1.70	1.22	2.39	0.002
Prior surgery for UTUC								
No	Reference			—	Reference			—
Yes	1.11	0.79	1.57	0.539	1.42	0.98	2.07	0.066
Neoadjuvant chemotherapy								
Yes	Reference			—	Reference			—
No	0.93	0.76	1.12	0.458	1.19	0.94	1.52	0.143
eGFR								
>60	Reference			—	Reference			—
30–60	1.22	1.00	1.50	0.053	1.21	0.97	1.50	0.097
<30	1.15	0.78	1.71	0.481	0.94	0.62	1.44	0.792
Histopathology of TURBT specimens								
Urothelial carcinoma	Reference			—	Reference			—
Urothelial carcinoma with variant	1.21	0.92	1.60	0.174	1.11	0.83	1.49	0.489
Other histology	1.42	0.90	2.23	0.129	1.38	0.87	2.20	0.175
Clinical T stage								
<=1	Reference			—	Reference			—
2	1.65	1.23	2.23	0.001	1.92	1.39	2.66	<0.0001
>=3	2.65	1.96	3.57	<0.001	2.82	2.00	3.96	<0.0001
Clinical N stage								
0	Reference			—	Reference			—
> = 1	1.97	1.46	2.65	<0.001	1.67	1.21	2.30	0.002
Radical cystectomy procedure, *N* (%)								
Open	Reference			—	Reference			—
Laparoscopic	0.98	0.74	1.29	0.878	1.09	0.82	1.47	0.543
Robot‐assisted	0.99	0.80	1.23	0.944	1.13	0.89	1.44	0.304
Lymph node dissection								
Yes	Reference			—	Reference			—
No	1.28	0.94	1.74	0.114	1.24	0.89	1.74	0.208
Fib‐4 index								
<3.5	Reference			—				
>=3.5	1.18	0.76	1.86	0.461				
ALBI score								
<=−2.6	Reference			—				
>−2.6	1.32	1.09	1.61	0.005				
NLR								
<5.0	Reference			—				
>=5.0	1.96	1.50	2.55	<0.001				
FAN score								
<=1	Reference			—	Reference			
>=2	2.22	1.66	2.96	<0.001	2.12	1.55	2.91	<0.0001

Abbreviations: ALBI, albumin–bilirubin; ECOG PS, Eastern Cooperative Oncology Group performance status; eGFR, estimated glomerular filtration rate; Fib‐4, fibrosis‐4; GC, Gemcitabine Cisplatin; GN, Gemcitabine Nedaplatin; NLR, neutrophil–lymphocyte ratio.

FAN score ≥2 was also independently associated with poorer CSS (HR 2.80, 95% CI 2.00–3.92; *p* < 0.0001). Other independent predictors of worse CSS included lower BMI ((HR 0.96 per unit, 95% CI 0.92–0.99; *p* = 0.013), higher clinical T stage (cT2: HR 2.16, 95% CI 1.45–3.21; ≥cT3: HR 3.57, 95% CI 2.37–5.37; both *p* < 0.0001) and higher clinical N stage (HR 1.84, 95% CI 1.30–2.61; *p* = 0.001) (Table S2).

For OS, FAN score ≥2 remained an independent adverse factor (HR 2.70, 95% CI 2.03–3.59; *p* < 0.0001). Additional independent predictors of worse OS were higher age (HR 1.02 per year, 95% CI 1.00–1.03; *p* = 0.024), male sex (reference female; female HR 0.71, 95% CI 0.56–0.90; *p* = 0.005), lower BMI (HR 0.95 per unit, 95% CI 0.92–0.98; *p* = 0.001), prior UTUC surgery (HR 1.52, 95% CI 1.07–2.16; *p* = 0.021), omission of neoadjuvant chemotherapy (HR 1.30, 95% CI 1.02–1.64; *p* = 0.033), higher clinical T stage (cT2: HR 1.65, 95% CI 1.22–2.24; ≥cT3: HR 2.37, 95% CI 1.73–3.26; both p ≤ 0.001), clinical N stage (HR 1.77, 95% CI 1.29–2.43; *p* < 0.0001) and omission of lymph node dissection at RC (HR 1.49, 95% CI 1.11–2.01; *p* = 0.008) (Table 3).

**TABLE 3:** Univariable and multivariable Cox regression analysis of OS.

	Univariable				Multivariable			
	HR	95% CI		*P*‐value	HR	95% CI		*P‐*value
		Lower	Higher			Lower	Higher	
Age								
Continuous (/1 year)	1.03	1.01	1.04	<0.0001	1.02	1.00	1.03	0.024
Gender								
Male	Reference			—	Reference			—
Female	0.83	0.66	1.04	0.107	0.71	0.56	0.90	0.005
BMI								
Continuous (/1 kg/m^2^)	0.95	0.92	0.98	0.001	0.95	0.92	0.98	0.001
ECOG PS								
0	Reference			—	Reference			—
1	0.99	0.80	1.21	0.894	0.84	0.68	1.04	0.109
>=2	1.74	1.26	2.39	0.001	1.31	0.94	1.83	0.110
Prior surgery for UTUC								
No	Reference			—	Reference			—
Yes	1.37	1.00	1.88	0.051	1.52	1.07	2.16	0.021
Neoadjuvant chemotherapy								
Yes	Reference			—	Reference			—
No	1.12	0.93	1.36	0.223	1.30	1.02	1.64	0.033
eGFR								
>60	Reference			—	Reference			—
30–60	1.27	1.04	1.56	0.019	1.21	0.97	1.51	0.088
<30	1.82	1.29	2.58	0.001	1.28	0.88	1.88	0.195
Histopathology of TURBT specimens								
Urothelial carcinoma	Reference			—	Reference			—
Urothelial carcinoma with variant	1.14	0.87	1.51	0.334	1.06	0.80	1.41	0.691
Other histology	1.25	0.80	1.97	0.329	1.20	0.75	1.91	0.442
Clinical T stage								
<=1	Reference			—	Reference			—
2	1.18	0.90	1.56	0.231	1.65	1.22	2.24	0.001
>=3	1.98	1.51	2.61	<0.0001	2.37	1.73	3.26	<0.0001
Clinical N stage								
0	Reference			—	Reference			—
>=1	1.83	1.37	2.46	<0.0001	1.77	1.29	2.43	<0.0001
Radical cystectomy procedure, *N* (%)								
Open	Reference			—	Reference			—
Laparoscopic	1.11	0.86	1.44	0.407	1.29	0.98	1.70	0.073
Robot‐assisted	0.98	0.78	1.24	0.894	1.16	0.90	1.49	0.241
Lymph node dissection								
Yes	Reference			—	Reference			—
No	1.71	1.31	2.24	<0.0001	1.51	1.12	2.03	0.007
Fib‐4 index								
<3.5	Reference			—				
>=3.5	1.73	1.17	2.55	0.006				
ALBI score								
<=−2.6	Reference			—				
>−2.6	1.66	1.37	2.01	<0.0001				
NLR								
<5.0	Reference			—				
>=5.0	2.14	1.65	2.76	<0.0001				
FAN score								
<=1	Reference			—	Reference			
>=2	3.13	2.41	4.07	<0.0001	2.70	2.03	3.59	<0.0001

Abbreviations: ALBI, albumin–bilirubin; ECOG PS, Eastern Cooperative Oncology Group performance status; eGFR, estimated glomerular filtration rate; Fib‐4, fibrosis‐4; GC, Gemcitabine Cisplatin; GN, Gemcitabine Nedaplatin; NLR neutrophil–lymphocyte ratio.

We assessed Harrell's concordance index for multivariable models including either the composite FAN score or its individual components (Fib‐4 index, ALBI score and NLR) (Table S3). For RFS, the C‐index was 0.627 for the FAN score model and 0.629 for the component‐based model. For OS, both models demonstrated identical C‐indices (0.665). For CSS, the C‐index was 0.669 for the FAN score model and 0.673 for the component‐based model. These differences were minimal and do not suggest a clinically meaningful improvement with the component‐based models, supporting the use of the composite FAN score as a practical prognostic tool.

### 3.5 Prognostic stratification by FAN score

**FIGURE 1:**
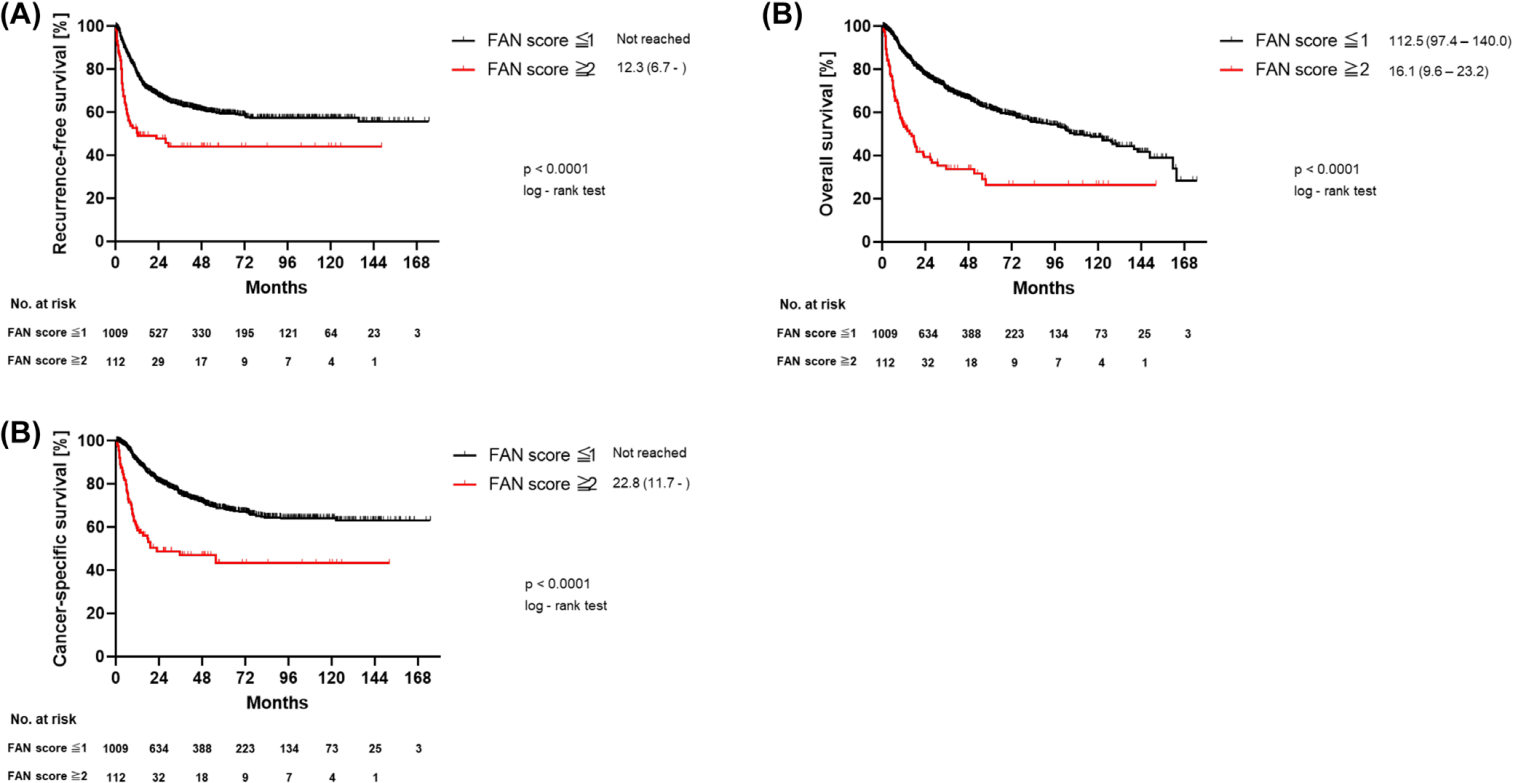
Kaplan–Meier curves for recurrence‐free survival (A), cancer‐specific survival (B) and overall survival (C) stratified by FAN score in the primary cohort. The numbers next to the legend indicate the median and 95% confidence interval.

Figure 1 presents RFS, CSS and OS curves for patients with FAN score ≤1 (*n* = 1009) versus FAN score ≥2 (*n* = 112). Patients with FAN ≥2 had significantly shorter RFS, CSS and OS than those with FAN ≤1 (all *p* < 0.0001). Median RFS was not reached for FAN ≤1 and was 12.3 months (95% CI 6.7–not reached) for FAN ≥2. Median CSS was not reached for FAN ≤1 and was 22.8 months (95% CI 11.7–not reached) for FAN ≥2. Median OS was 112.5 months (95% CI 97.4–140.0) for FAN ≤1 and 16.1 months (95% CI 9.6–23.2) for FAN ≥2. When stratified into four groups (FAN 0, 1, 2 and 3), prognosis worsened with increasing FAN score; a marked difference was observed between FAN 1 and FAN 2 (Figure S2). In an analysis restricted to patients with clinical T2 disease, Kaplan–Meier curves stratified by FAN score similarly demonstrated significantly worse RFS, CSS and OS in patients with a FAN score ≥2 compared with those with a FAN score ≤1 (Figure S3).

### 3.6 Independent cohort

**FIGURE 2:**
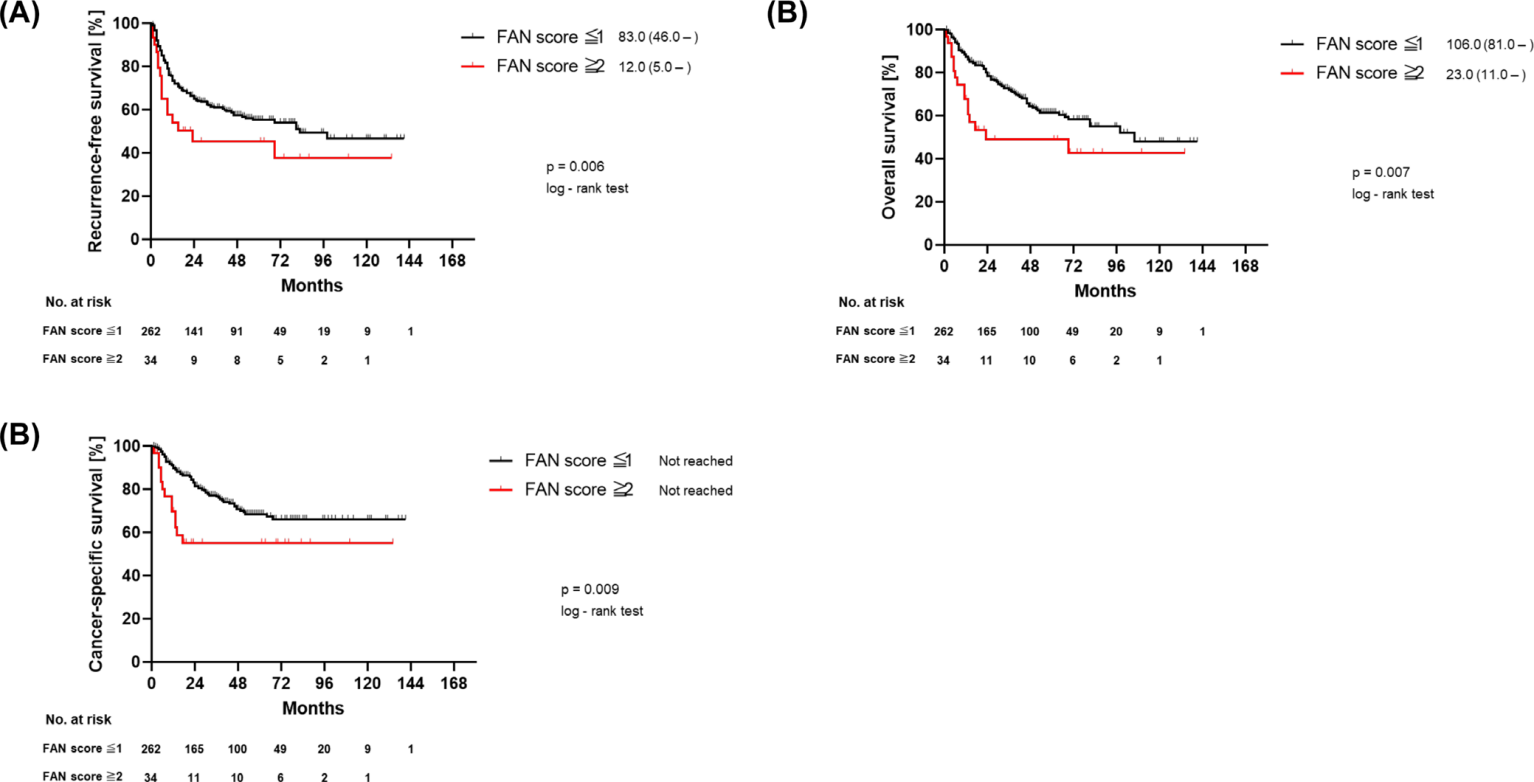
Kaplan–Meier curves for recurrence‐free survival (A), cancer‐specific survival (B) and overall survival (C) stratified by FAN score in the independent cohort. The numbers next to the legend indicate the median and 95% confidence interval.

Patient characteristics for the independent cohort (*n* = 296) are summarised in Table S4. Thirty‐four patients (11.4%) had FAN score ≥2, a proportion similar to the primary cohort. Kaplan–Meier curves in Figure 2 show that FAN ≥2 was associated with significantly poorer RFS, CSS and OS compared with FAN ≤1, demonstrating consistent prognostic stratification in this independent cohort.

## 4 DISCUSSION

In this multicentre cohort, the FAN score—previously validated as a prognostic marker in metastatic urothelial carcinoma treated with platinum chemotherapy and pembrolizumab—also stratified outcomes for patients undergoing RC. The prognostic value of a FAN score ≥2 was confirmed in both the primary cohort of 1121 patients and an independent cohort, supporting the score's reproducibility and potential clinical utility. To our knowledge, this is the first study to demonstrate the FAN score's usefulness as a preoperative prognostic predictor for patients undergoing surgery for bladder cancer. The FAN score offers practical advantages as a preoperative marker because it can be calculated from routine laboratory tests without additional cost or invasive procedures. A FAN score ≥2 predicted worse RFS, CSS and OS independently of clinical TNM stage, ECOG PS and receipt of neoadjuvant chemotherapy. These findings suggest that the FAN score captures aspects of patient condition—systemic inflammation, nutritional status, liver function and immune competence—that are not fully represented by traditional tumour‐centric variables.^17^, ^18^ Of note, ECOG PS, although a well‐recognised prognostic indicator, lost significance in multivariable models that included the FAN score. Several explanations are plausible. First, components of the FAN score (particularly NLR and ALBI) may reflect nutritional decline and frailty that overlap with the information conveyed by ECOG PS, introducing confounding and leaving the objectively measured FAN score as the stronger independent predictor. Second, PS is subject to interobserver variability across institutions, whereas the FAN score is derived from objective laboratory values and may therefore be more reproducible. Third, the relatively small proportion of patients with poor PS in our cohort could have limited power to demonstrate an independent effect. Collectively, these observations indicate that the FAN score may act as a robust and objective surrogate for global patient condition in the preoperative setting.

The biological significance of the FAN score is also noteworthy. Elevated NLR reflects systemic inflammation and immunosuppression, which are associated with tumour progression and treatment resistance.^19^ The Fib‐4 index and ALBI score were originally developed as markers of liver fibrosis and liver function, but they may also reflect systemic metabolic and inflammatory conditions that affect cancer outcomes.^20^, ^21^ Therefore, the FAN score may serve as a surrogate marker for the interplay between tumour biology and host response.

This study has several limitations. First, its retrospective design introduces potential selection bias, and perioperative management (including use of neoadjuvant chemotherapy and the extent of lymph node dissection) was not standardised across centres. Second, data for some established prognostic variables such as anaemia and hydronephrosis were not available and could not be included in the analyses. In addition, information on postoperative adjuvant chemotherapy was not uniformly available across institutions and therefore could not be included in the multivariable analyses. Third, only approximately 10% of patients had a FAN score ≥2, which limits subgroup sample size and may affect the precision of estimates for this high‐risk group. Importantly, although a FAN score ≥2 identified patients with poorer median survival, these patients still included individuals with meaningful survival (median OS 16.1 months in the primary cohort and 23.0 months in the independent cohort), indicating that a high FAN score alone should not be used to deny standard surgical therapy. Future prospective studies should evaluate whether the FAN score can guide preoperative decision making, perioperative management or selection for alternative or additional therapies (e.g., intensified systemic therapy or multimodal approaches) in patients at high risk. Investigation of the underlying biological mechanisms linking the FAN components to tumour progression may also clarify opportunities for targeted interventions.

## 5 CONCLUSION

The preoperative FAN score, consisting of the fibrosis‐4 index, ALBI score and NLR, is a prognostic predictor for bladder cancer patients undergoing RC.

## AUTHOR CONTRIBUTIONS

Study concept and design: Kawashima. Acquisition of data: Kawashima, Ujike, Takao, Miyake, Osaki, Horitani, Iwanishi, Takada, Oki, Yoshida, Matsushita, Kakimoto, Nagahara, Yamashita. Analysis and interpretation of data: Ishizuya, Kawashima, Yamashita. Drafting of the manuscript: Ishizuya. Critical revision of the manuscript for important intellectual content: Ishizuya, Kawashima, Hayashi, Yamamoto, Kato, Hatano. Statistical analysis: Ishizuya, Yamashita. Administrative, technical, or material support: Kawashima. Supervision: Kohjimoto, Hara, Nonomura. Others: None.

## CONFLICT OF INTEREST STATEMENT

The authors declare no competing interests.

## Supporting information


**Figure S1.** Kaplan–Meier curves for recurrence‐free survival (A), cancer‐specific survival (B) and overall survival (C) in the primary cohort. The numbers next to the legend indicate the median and 95% confidence interval.
**Figure S2.** Kaplan–Meier curves for recurrence‐free survival (A), cancer‐specific survival (B) and overall survival (C) stratified by FAN score in the primary cohort. The numbers next to the legend indicate the median and 95% confidence interval.
**Figure S3.** Kaplan–Meier curves for recurrence‐free survival (A), cancer‐specific survival (B) and overall survival (C) stratified by FAN score in patients with clinical T2 disease in the primary cohort. The numbers next to the legend indicate the median and 95% confidence interval.

**Table S1.** Comparison of clinicopathological factors between the FAN score groups.
**Table S2.** Univariable and multivariable Cox regression analysis of CSS.
**Table S3.** Multivariable Cox regression analyses including the individual components of the FAN score for RFS, CSS and OS.
**Table S4.** Patient characteristics of the independent cohort.

## Data Availability

Detailed data were generated at Osaka University. Derived data supporting the results of this study are available upon request from the corresponding author.
