# Supplementary material for: Preoperative FAN score predicts survival outcomes after radical cystectomy for bladder cancer
Source: BJUI Compass. 2026 Apr 30;7(5):e70221. doi: 10.1002/bco2.70221 (PMC13129595; doi:10.1002/bco2.70221)

Supplementary Figure 1

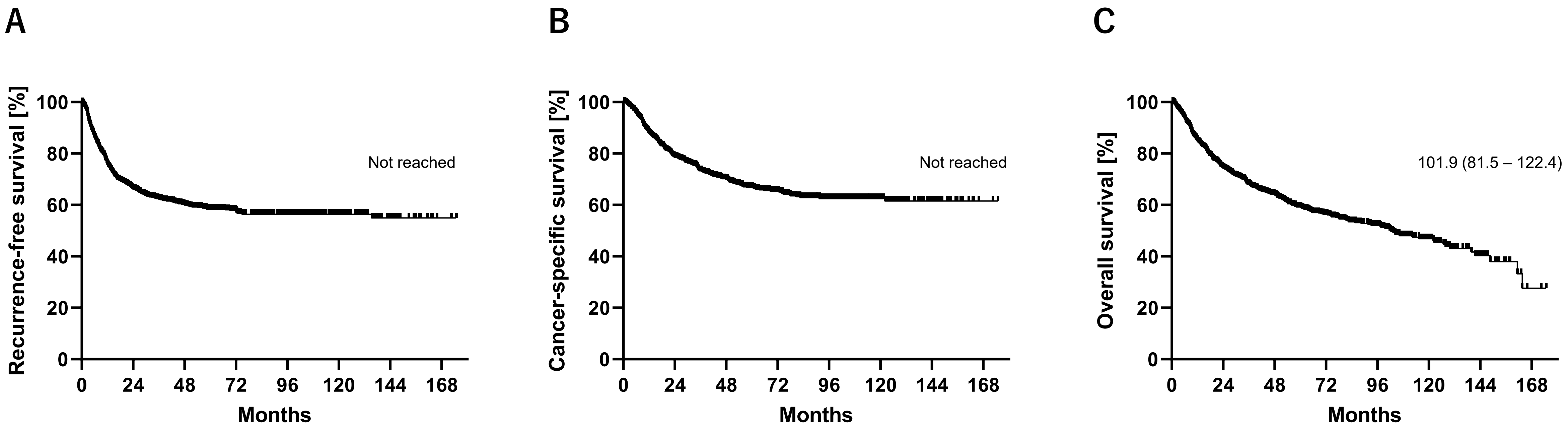

**No. at risk**	1121	557	347	204	128	68	24	3
**No. at risk**	1121	665	406	232	141	77	26	3
**No. at risk**	1121	665	406	232	141	77	26	3

Supplementary Figure 2

A

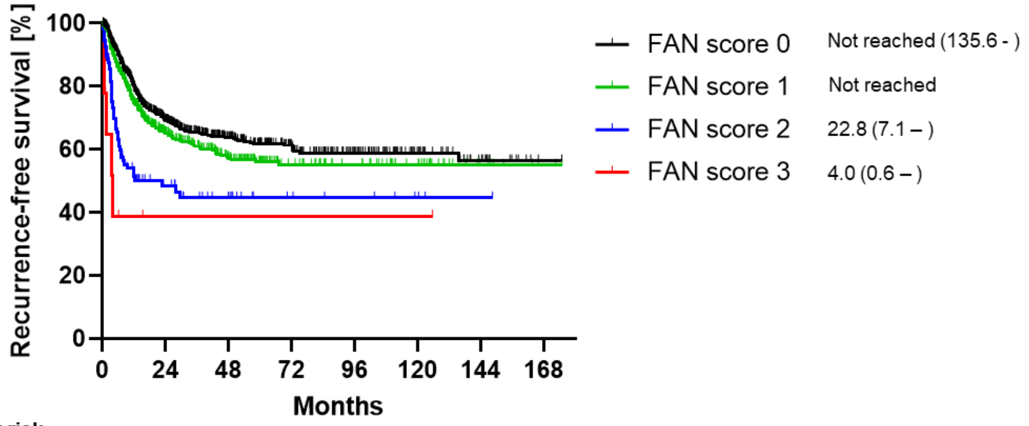

No. at risk

FAN score 0	600	336	208	129	76	36	15	1
FAN score 1	409	191	122	66	45	28	8	2
FAN score 2	103	28	16	8	6	3	1	
FAN score 3	9	1	1	1	1	1		

C

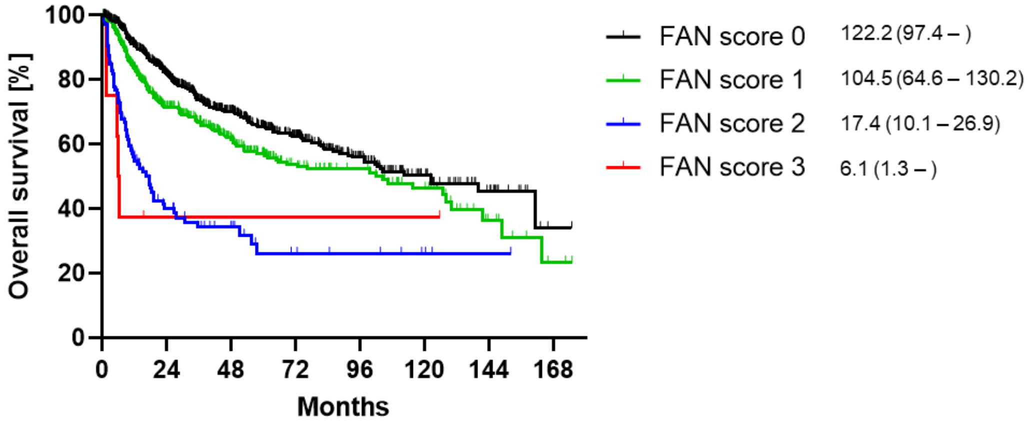

No. at risk

FAN score 0	600	407	248	150	84	41	16	1
FAN score 1	409	225	140	73	50	32	9	2
FAN score 2	103	31	17	8	6	3	1	
FAN score 3	9	1	1	1	1	1		

B

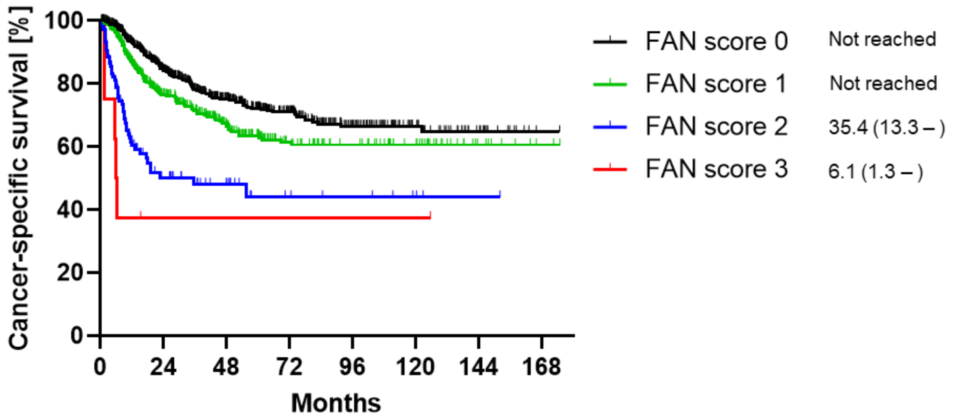

No. at risk

FAN score 0	600	407	248	150	84	41	16	1
FAN score 1	409	225	140	73	50	32	9	2
FAN score 2	103	31	17	8	6	3	1	
FAN score 3	9	1	1	1	1	1		

Supplementary Figure 3

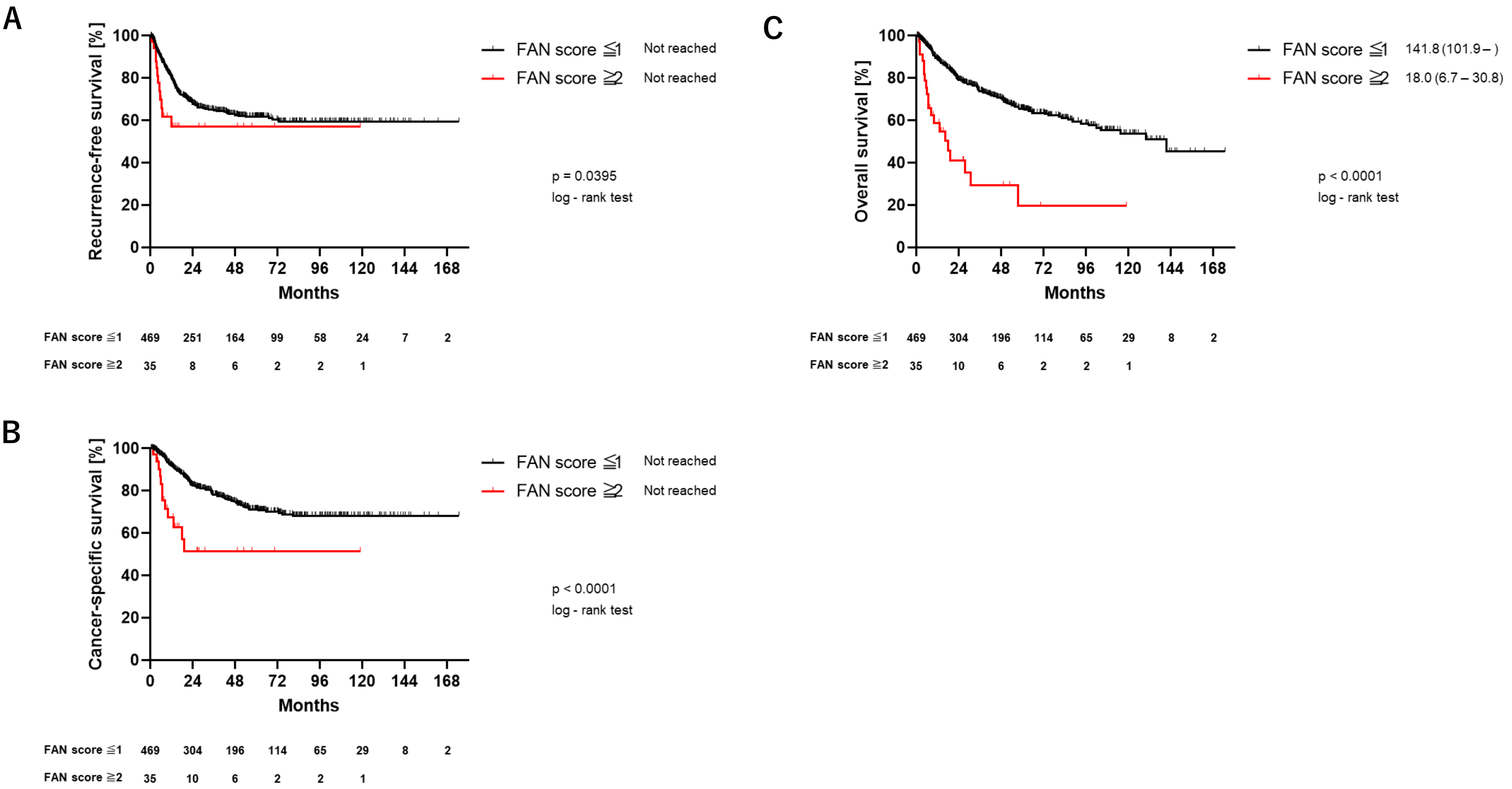

Supplement: Supplementary file 1 — Figure S1. Kaplan–Meier curves for recurrence‐free survival (A), cancer‐specific survival (B) and overall survival (C) in the primary cohort. The numbers next to the legend indicate the median and 95% confidence interval. Figure S2. Kaplan–Meier curves for recurrence‐free survival (A), cancer‐specific survival (B) and overall survival (C) stratified by FAN score in the primary cohort. The numbers next to the legend indicate the median and 95% confidence interval. Figure S3. Kaplan–Meier curves for recurrence‐free survival (A), cancer‐specific survival (B) and overall survival (C) stratified by FAN score in patients with clinical T2 disease in the primary cohort. The numbers next to the legend indicate the median and 95% confidence interval. [file BCO2-7-e70221-s001.pdf]
