# Supplementary material for: Preoperative FAN score predicts survival outcomes after radical cystectomy for bladder cancer
Source: BJUI Compass. 2026 Apr 30;7(5):e70221. doi: 10.1002/bco2.70221 (PMC13129595; doi:10.1002/bco2.70221)
Supplement: Supplementary file 2 — Table S1. Comparison of clinicopathological factors between the FAN score groups. Table S2. Univariable and multivariable Cox regression analysis of CSS. Table S3. Multivariable Cox regression analyses including the individual components of the FAN score for RFS, CSS and OS. Table S4. Patient characteristics of the independent cohort. [file BCO2-7-e70221-s002.docx]

**Supplemental Table 1. Comparison of clinicopathological factors between the FAN score groups**

| **Characteristic** | **FAN score 0,1 (n=1009)** | | **FAN score 2,3 (n=112)** | | **p value** |
| --- | --- | --- | --- | --- | --- |
| **Age, years, range, (median)** | 29 - 90 | 72.7 | 45 - 89 | 75.9 | <0.001 |
| **Gender, N (%)** | | | | | |
| Male | 762 | 75.5 | 85 | 75.9 | 0.926 |
| Female | 247 | 24.5 | 27 | 24.1 |  |
| **BMI, kg/m^2^, range, (median)** | 13.4 - 38.5 | 22.0 | 15.5 - 34.4 | 21.5 | 0.527 |
| **ECOG PS, N (%)** | | | | | |
| 0 | 501 | 49.7 | 33 | 29.5 | <0.001 |
| 1 | 423 | 41.9 | 48 | 42.9 |  |
| ≥2 | 85 | 8.4 | 31 | 27.7 |  |
| **Neoadjuvant chemotherapy, N (%)** | | | | | |
| No | 515 | 51.0 | 75 | 67.0 | 0.001 |
| Yes | 494 | 49.0 | 37 | 33.0 |  |
| **eGFR (ml/min/1.73 m2), range, (median)** | 3 - 176 | 57 | 3 - 116 | 50 | 0.001 |
| **Fib-4 index, N (%)** | | |  |  |  |
| < 3.5 | 988 | 97.9 | 75 | 67.0 | <0.001 |
| ≥ 3.5 | 21 | 2.1 | 37 | 33.0 |  |
| **ALBI score, N (%)** | | | | | |
| ≤ − 2.6 | 675 | 66.9 | 0 | 0.0 | <0.001 |
| ＞ − 2.6 | 334 | 33.1 | 112 | 100.0 |  |
| **NLR, N (%)** | | | | | |
| < 5.0 | 955 | 94.6 | 28 | 25.0 | <0.001 |
| ≥ 5.0 | 54 | 5.4 | 84 | 75.0 |  |
| **Histopathology of TURBT specimens, N (%)** | | | | | |
| Urothelial carcinoma | 842 | 83.4 | 89 | 79.5 | 0.565 |
| Urothelial carcinoma with variant | 129 | 12.8 | 18 | 16.1 |  |
| Other histology | 38 | 3.8 | 5 | 4.5 |  |
| **Clinical T stage, N (%)** | | | | | |
| <=1 | 235 | 23.3 | 25 | 22.3 | 0.001 |
| 2 | 469 | 46.5 | 35 | 31.3 |  |
| >=3 | 305 | 30.2 | 52 | 46.4 |  |
| **Clinical N stage, N (%)** | | | | | |
| 0 | 926 | 91.8 | 105 | 93.8 | 0.583 |
| >=1 | 83 | 8.2 | 7 | 6.3 |  |

ECOG PS Eastern Cooperative Oncology Group performance status, eGFR estimated Glomerular Filtration Rate, Fib-4 Fibrosis-4, ALBI albumin–bilirubin, NLR neutrophil-lymphocyte ratio, TURBT transurethral resection of bladder tumor

**Supplemental Table 2. Univariable and multivariable Cox regression analysis of CSS**

|  | **Univariable** | | | | **Multivariable** | | | |
| --- | --- | --- | --- | --- | --- | --- | --- | --- |
|  | **HR** | **95%CI** | | **P value** | **HR** | **95%CI** | | **P value** |
|  |  | **Lower** | **Higher** |  |  | **Lower** | **Higher** |  |
| **Age** |  |  |  |  |  |  |  |  |
| continuous (/1year) | 1.01 | 1.00 | 1.02 | 0.133 | 1.00 | 0.99 | 1.01 | 0.981 |
| **Gender** |  |  |  |  |  |  |  |  |
| Male | Reference |  |  | - | Reference |  |  | - |
| Female | 0.93 | 0.58 | 0.71 | 1.207 | 0.78 | 0.60 | 1.03 | 0.081 |
| **BMI** |  |  |  |  |  |  |  |  |
| continuous (/1 kg/m^2^) | 0.96 | 0.92 | 0.99 | 0.010 | 0.96 | 0.92 | 0.99 | 0.013 |
| **ECOG PS** |  |  |  |  |  |  |  |  |
| 0 | Reference |  |  | - | Reference |  |  | - |
| 1 | 0.96 | 0.76 | 1.23 | 0.758 | 0.87 | 0.68 | 1.12 | 0.286 |
| >=2 | 1.66 | 1.14 | 2.42 | 0.009 | 1.34 | 0.90 | 2.00 | 0.153 |
| **Prior surgery for UTUC** |  |  |  |  |  |  |  |  |
| No | Reference |  |  | - | Reference |  |  | - |
| Yes | 1.15 | 0.77 | 1.70 | 0.501 | 1.41 | 0.92 | 2.18 | 0.118 |
| **Neoadjuvant chemotherapy** |  |  |  |  |  |  |  |  |
| Yes | Reference |  |  | - | Reference |  |  | - |
| No | 0.97 | 0.78 | 1.22 | 0.798 | 1.31 | 1.00 | 1.71 | 0.051 |
| **eGFR** |  |  |  |  |  |  |  |  |
| > 60 | Reference |  |  | - | Reference |  |  | - |
| 30 - 60 | 1.31 | 1.03 | 1.66 | 0.025 | 1.33 | 1.03 | 1.72 | 0.028 |
| < 30 | 1.47 | 0.95 | 2.28 | 0.083 | 1.13 | 0.70 | 1.81 | 0.619 |
| **Histopathology of TURBT specimens** |  |  |  |  |  |  |  |  |
| Urothelial carcinoma | Reference |  |  | - | Reference |  |  | - |
| Urothelial carcinoma with variant | 1.40 | 1.03 | 1.89 | 0.032 | 1.28 | 0.93 | 1.76 | 0.129 |
| Other histology | 1.46 | 0.88 | 2.42 | 0.144 | 1.34 | 0.80 | 2.27 | 0.267 |
| **Clinical T stage** |  |  |  |  |  |  |  |  |
| <=1 | Reference |  |  | - | Reference |  |  | - |
| 2 | 1.66 | 1.15 | 2.40 | 0.007 | 2.16 | 1.45 | 3.21 | <0.001 |
| >=3 | 3.18 | 2.22 | 4.57 | <.0001 | 3.57 | 2.37 | 5.37 | <.0001 |
| **Clinical N stage** |  |  |  |  |  |  |  |  |
| 0 | Reference |  |  | - | Reference |  |  | - |
| >=1 | 2.16 | 1.57 | 2.99 | <.0001 | 1.84 | 1.30 | 2.61 | 0.001 |
| **Radical Cystectomy Procedure, N (%)** | |  |  |  |  |  |  |  |
| Open | Reference |  |  | - | Reference |  |  | - |
| Laparoscopic | 1.11 | 0.82 | 1.49 | 0.513 | 1.32 | 0.96 | 1.82 | 0.093 |
| Robot-assisted | 1.01 | 0.78 | 1.31 | 0.945 | 1.23 | 0.93 | 1.64 | 0.149 |
| **Lymph node dissection** |  |  |  |  |  |  |  |  |
| Yes | Reference |  |  | - | Reference |  |  | - |
| No | 1.47 | 1.05 | 2.05 | 0.026 | 1.35 | 0.93 | 1.97 | 0.115 |
| **Fib-4 index** |  |  |  |  |  |  |  |  |
| < 3.5 | Reference |  |  | - |  |  |  |  |
| >= 3.5 | 1.54 | 0.96 | 2.48 | 0.076 |  |  |  |  |
| **ALBI score** |  |  |  |  |  |  |  |  |
| <= -2.6 | Reference |  |  | - |  |  |  |  |
| >−2.6 | 1.69 | 1.35 | 2.12 | <.0001 |  |  |  |  |
| **NLR** |  |  |  |  |  |  |  |  |
| < 5.0 | Reference |  |  | - |  |  |  |  |
| >=5.0 | 2.32 | 1.73 | 3.11 | <.0001 |  |  |  |  |
| **FAN score** |  |  |  |  |  |  |  |  |
| <=1 | Reference |  |  | - | Reference |  |  |  |
| >=2 | 3.09 | 2.27 | 4.20 | <.0001 | 2.80 | 2.00 | 3.92 | <.0001 |

ECOG PS Eastern Cooperative Oncology Group performance status, eGFR estimated Glomerular Filtration Rate, GC Gemcitabine Cisplatin, GN Gemcitabine Nedaplatin, Fib-4 Fibrosis-4, ALBI albumin–bilirubin, NLR neutrophil-lymphocyte ratio

**Supplemental Table 3. Supplementary Table 3. Multivariable Cox regression analyses including the individual components of the FAN score for RFS, CSS, and OS.**

|  | **RFS** | | | | **CSS** | | | | **OS** | | | |
| --- | --- | --- | --- | --- | --- | --- | --- | --- | --- | --- | --- | --- |
|  | **HR** | **95%CI** | | **P value** | **HR** | **95%CI** | | **P value** | **HR** | **95%CI** | | **P value** |
|  |  | **Low** | **High** |  |  | **Low** | **High** |  |  | **Low** | **High** |  |
| **Age** |  |  |  |  |  |  |  |  |  |  |  |  |
| continuous (/1year) | 0.99 | 0.98 | 1.01 | 0.483 | 1.00 | 0.98 | 1.01 | 0.818 | 1.01 | 1.00 | 1.03 | 0.041 |
| **Gender** |  |  |  |  |  |  |  |  |  |  |  |  |
| Male | Ref |  |  | - | Ref |  |  | - | Ref |  |  | - |
| Female | 0.85 | 0.67 | 1.07 | 0.165 | 0.81 | 0.61 | 1.06 | 0.125 | 0.73 | 0.57 | 0.92 | 0.009 |
| **BMI** |  |  |  |  |  |  |  |  |  |  |  |  |
| continuous (/1 kg/m^2^) | 0.97 | 0.94 | 1.00 | 0.088 | 0.96 | 0.93 | 1.00 | 0.031 | 0.96 | 0.93 | 0.99 | 0.004 |
| **ECOG PS** |  |  |  |  |  |  |  |  |  |  |  |  |
| 0 | Ref |  |  | - | Ref |  |  | - | Ref |  |  | - |
| 1 | 1.01 | 0.81 | 1.25 | 0.950 | 0.86 | 0.67 | 1.11 | 0.238 | 0.83 | 0.67 | 1.03 | 0.099 |
| >=2 | 1.67 | 1.19 | 2.35 | 0.003 | 1.29 | 0.86 | 1.92 | 0.215 | 1.28 | 0.92 | 1.79 | 0.146 |
| **Prior surgery for UTUC** |  |  |  |  |  |  |  |  |  |  |  |  |
| No | Ref |  |  | - | Ref |  |  | - | Ref |  |  | - |
| Yes | 1.37 | 0.94 | 1.99 | 0.100 | 1.37 | 0.89 | 2.11 | 0.156 | 1.47 | 1.03 | 2.09 | 0.033 |
| **Neoadjuvant chemotherapy** |  |  |  |  |  |  |  |  |  |  |  |  |
| Yes | Ref |  |  | - | Ref |  |  | - | Ref |  |  | - |
| No | 1.18 | 0.93 | 1.49 | 0.191 | 1.30 | 0.99 | 1.70 | 0.061 | 1.29 | 1.02 | 1.64 | 0.033 |
| **eGFR** |  |  |  |  |  |  |  |  |  |  |  |  |
| > 60 | Ref |  |  | - | Ref |  |  | - | Ref |  |  | - |
| 30 - 60 | 1.17 | 0.94 | 1.46 | 0.160 | 1.27 | 0.99 | 1.64 | 0.064 | 1.16 | 0.93 | 1.44 | 0.188 |
| < 30 | 0.96 | 0.63 | 1.48 | 0.863 | 1.12 | 0.69 | 1.80 | 0.653 | 1.28 | 0.87 | 1.87 | 0.205 |
| **Histopathology of TURBT specimens** |  |  |  |  |  |  |  |  |  |  |  |  |
| Urothelial carcinoma | Ref |  |  | - | Ref |  |  | - | Ref |  |  | - |
| Urothelial carcinoma with variant | 1.08 | 0.80 | 1.44 | 0.613 | 1.26 | 0.91 | 1.73 | 0.160 | 1.06 | 0.79 | 1.41 | 0.711 |
| Other histology | 1.37 | 0.86 | 2.18 | 0.185 | 1.32 | 0.78 | 2.23 | 0.294 | 1.17 | 0.73 | 1.86 | 0.515 |
| **Clinical T stage** |  |  |  |  |  |  |  |  |  |  |  |  |
| <=1 | Ref |  |  | - | Ref |  |  | - | Ref |  |  | - |
| 2 | 1.87 | 1.35 | 2.59 | <.0001 | 2.10 | 1.41 | 3.12 | <0.001 | 1.60 | 1.18 | 2.18 | 0.003 |
| >=3 | 2.76 | 1.96 | 3.89 | <.0001 | 3.45 | 2.29 | 5.19 | <.0001 | 2.31 | 1.68 | 3.18 | <.0001 |
| **Clinical N stage** |  |  |  |  |  |  |  |  |  |  |  |  |
| 0 | Ref |  |  | - | Ref |  |  | - | Ref |  |  | - |
| >=1 | 1.63 | 1.19 | 2.25 | 0.003 | 1.84 | 1.30 | 2.62 | 0.001 | 1.76 | 1.28 | 2.43 | <.0001 |
| **Radical Cystectomy Procedure, N (%)** |  |  |  |  |  |  |  |  |  |  |  |  |
| Open | Ref |  |  | - | Ref |  |  | - | Ref |  |  | - |
| Laparoscopic | 1.09 | 0.81 | 1.46 | 0.558 | 1.30 | 0.94 | 1.81 | 0.108 | 1.29 | 0.98 | 1.71 | 0.073 |
| Robot-assisted | 1.12 | 0.88 | 1.42 | 0.357 | 1.21 | 0.91 | 1.62 | 0.181 | 1.16 | 0.90 | 1.49 | 0.253 |
| **Lymph node dissection** |  |  |  |  |  |  |  |  |  |  |  |  |
| Yes | Ref |  |  | - | Ref |  |  | - | Ref |  |  | - |
| No | 1.24 | 0.88 | 1.73 | 0.219 | 1.33 | 0.91 | 1.93 | 0.139 | 1.48 | 1.10 | 2.00 | 0.010 |
| **Fib-4 index** |  |  |  |  |  |  |  |  |  |  |  |  |
| < 3.5 | Ref |  |  | - | Ref |  |  | - | Ref |  |  | - |
| >= 3.5 | 1.11 | 0.69 | 1.78 | 0.661 | 1.41 | 0.85 | 2.32 | 0.184 | 1.47 | 0.97 | 2.23 | 0.073 |
| **ALBI score** |  |  |  |  |  |  |  |  |  |  |  |  |
| <= -2.6 | Ref |  |  | - | Ref |  |  | - | Ref |  |  | - |
| >−2.6 | 1.21 | 0.98 | 1.49 | 0.079 | 1.49 | 1.17 | 1.89 | 0.001 | 1.38 | 1.12 | 1.69 | 0.002 |
| **NLR** |  |  |  |  |  |  |  |  |  |  |  |  |
| < 5.0 | Ref |  |  | - | Ref |  |  | - | Ref |  |  | - |
| >=5.0 | 1.72 | 1.30 | 2.28 | <.0001 | 1.88 | 1.38 | 2.57 | <.0001 | 1.75 | 1.33 | 2.30 | <.0001 |

ECOG PS Eastern Cooperative Oncology Group performance status, eGFR estimated Glomerular Filtration Rate, GC Gemcitabine Cisplatin, GN Gemcitabine Nedaplatin, Fib-4 Fibrosis-4, ALBI albumin–bilirubin, NLR neutrophil-lymphocyte ratio

**Supplemental Table 4. Patient characteristics of the independent cohort**

| **Characteristic** | **n=296** | |
| --- | --- | --- |
| **Age, years, range, (median)** | 43 - 90 | 73.0 |
| **Gender, N (%)** |  |  |
| Male | 236 | 79.7 |
| Female | 60 | 20.3 |
| **BMI, kg/m^2^, range, (median)** | 14.0 - 35.3 | 22.1 |
| **ECOG PS, N (%)** |  |  |
| 0 | 243 | 82.1 |
| 1 | 35 | 11.8 |
| ≥2 | 18 | 6.1 |
| **Prior surgery for UTUC, N (%)** |  |  |
| No | 282 | 95.3 |
| Yes | 14 | 4.7 |
| **Neoadjuvant chemotherapy, N (%)** |  |  |
| No | 204 | 68.9 |
| Yes | 92 | 31.1 |
| **eGFR (ml/min/1.73 m2), range, (median)** | 6 - 141 | 59 |
| **eGFR (ml/min/1.73 m2), N (%)** |  |  |
| > 60 | 141 | 47.6 |
| 30 - 60 | 136 | 45.9 |
| < 30 | 19 | 6.4 |
| **Fib-4 index, N (%)** | | |
| < 3.5 | 276 | 93.2 |
| ≥ 3.5 | 20 | 6.8 |
| **ALBI score, N (%)** |  |  |
| ≤ − 2.6 | 179 | 60.5 |
| ＞ − 2.6 | 117 | 39.5 |
| **NLR, N (%)** |  |  |
| < 5.0 | 267 | 90.2 |
| ≥ 5.0 | 29 | 9.8 |
| **FAN score, N (%)** |  |  |
| 0 | 165 | 55.7 |
| 1 | 97 | 32.8 |
| 2 | 33 | 11.1 |
| 3 | 1 | 0.3 |
| **Histopathology of TURBT specimens, N (%)** |  |  |
| Urothelial carcinoma | 249 | 84.1 |
| Urothelial carcinoma with variant | 25 | 8.4 |
| Other histology | 22 | 7.4 |
| **Clinical T stage, N (%)** |  |  |
| 0 | 2 | 0.7 |
| is | 26 | 8.8 |
| a | 2 | 0.7 |
| 1 | 22 | 7.4 |
| 2 | 145 | 49.0 |
| 3 | 75 | 25.3 |
| 4 | 24 | 8.1 |
| **Clinical N stage, N (%)** |  |  |
| 0 | 275 | 92.9 |
| 1 | 15 | 5.1 |
| 2 | 4 | 1.4 |
| 3 | 2 | 0.7 |
| **Radical Cystectomy Procedure, N (%)** |  |  |
| Open | 214 | 72.3 |
| Laparoscopic | 29 | 9.8 |
| Robot-assisted | 53 | 17.9 |
| **Lymph node dissection** |  |  |
| No | 2 | 0.7 |
| Yes | 294 | 99.3 |
| **Observation period, month, range (median)** | 0.5 - 141.0 | 33.0 |

ECOG PS Eastern Cooperative Oncology Group performance status, UTUC upper tract urothelial carcinoma, eGFR estimated Glomerular Filtration Rate, Fib-4 Fibrosis-4, ALBI albumin–bilirubin, NLR neutrophil-lymphocyte ratio, TURBT transurethral resection of bladder tumor
